# Post-traumatic missing medial malleolus: case report with a one-year follow-up

**DOI:** 10.1016/j.ijscr.2023.109083

**Published:** 2023-11-22

**Authors:** F. Lamnaouar, M.A. Kharroube, K. Tabbak, N. Nekeshima, A. Rajaallah, M. Rafai

**Affiliations:** Orthopedics and Traumatology Surgery at the 32 Pavilion of CHU Ibn Rochd of Casablanca, Morocco

**Keywords:** Ankle, Open fracture, Fracture dislocation, Missing medial malleolus, Osteoarthritis, Instability

## Abstract

**Introduction and importance:**

Ankle fractures result from a wide variety of mechanisms the biomechanics of this articulation make any fracture altering the contact of the articulation surface lead to increased stresses and an inevitable evolution to tibiotalar arthritis.

**Case presentation:**

We report the case of a 56-year-old patient admitted for open fracture dislocation of the ankle with a missing medial malleolus. He was treated by external fixation k-wiring and periosteal repair of the deltoid ligament. The one-year follow-up shows a good functional outcome with the patient’s satisfaction.

**Clinical discussion:**

In ankle fractures, some authors report no need for fixation of the medial malleolus. Furthermore, the absence of the medial malleolus is not necessarily accompanied by instability of the ankle; however, it inevitably leads to ankle osteoarthritis.

**Conclusion:**

Our patient admitted for a bimalleolar fracture with missing medial malleolus, as described in the literature, had a favorable evolution with a functional score that was judged fair.

## Highlights

• Medial malleolus considered long ago as the primary stabilizer of the ankle, is no longer taken as such.
• There is smilar functional results in the bimalleolar fracture, when the osteosynthesis of the medial malleolus is realized or not
• The cases report about missing medial malleolus objectifying good functional outcomes with natural evolution to osteoarthritis.

## 1 Introduction

Ankle fractures can result from a wide variety of mechanisms, with reported correlations between falls, jumps, trauma, and the occurrence of such injuries. Ankle fractures are associated with substantial morbidity, including posttraumatic arthritis [1].

The medial malleolus was universally accepted as being the primary ankle stabilizer. In the adult population, loss of the medial malleolus can lead to recurrent ankle instability [2] until 1977, when Yablon et al. [3] published a landmark study concluding that it is, in fact, the fibula that reliably guides and maintains talar reduction. The significance of the medial malleolus and supporting ligamentous structures has since been the subject of much clinical and biomechanical debate [4].

## 2 Case report

The reporting of this work follows the SCARE checklist criteria [11], ensuring adherence to guidelines for quality reporting in case series.

**Fig. 1:**
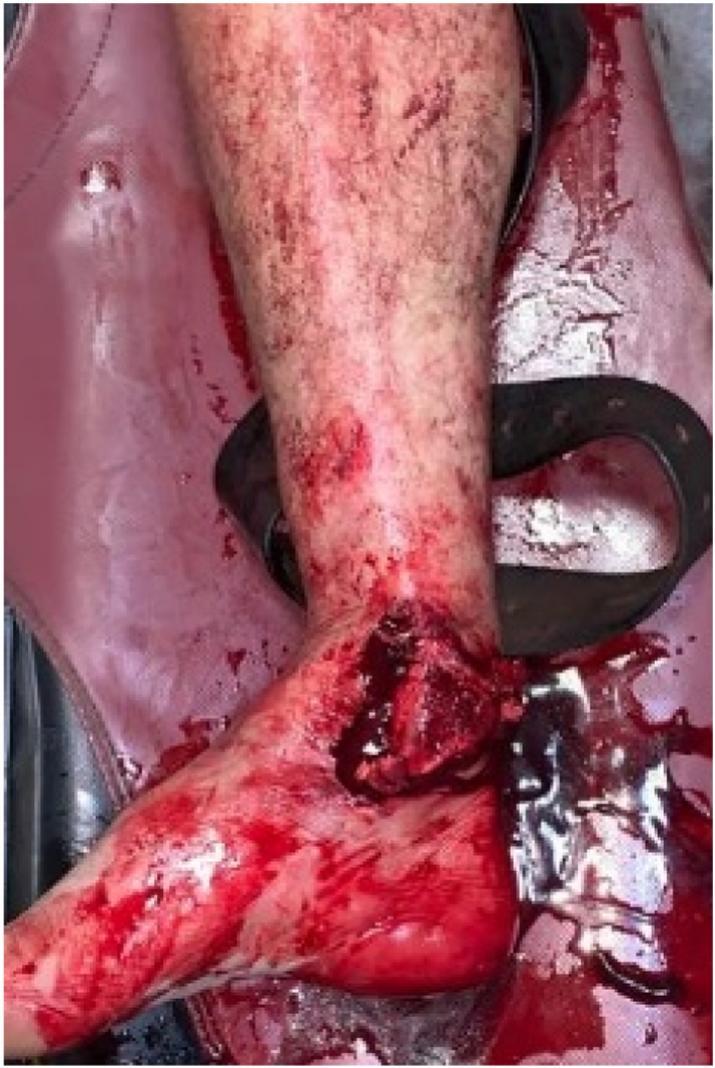
Initial presentation of the patient with open fracture-dislocation of the ankle with extrusion of the tibial mortise.

We report the case of a patient aged 56 years, an alcoholic who was a victim of a traffic accident that caused open trauma to the right ankle (Fig. 1).

**Fig. 2:**
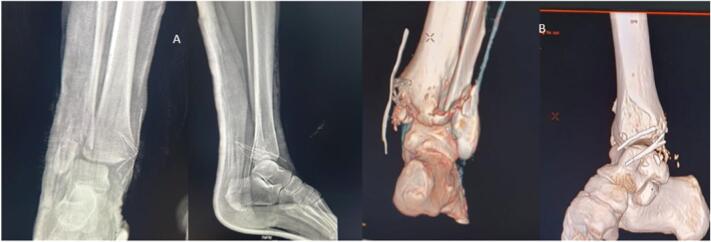
An X-ray image of the ankle after reduction with missing medial malleolus B: 3D image after the CT scan.

The patient was initially seen in the emergency department, and the physical examination found a patient who was in a state of drunkenness, with a stable hemodynamic and respiratory constant. There was an open luxation of the ankle, with the absence of the medial malleolus. The patient was scanned for associated lesions. X-ray imaging was performed for fracture luxation of the ankle (Fig. 2).

**Fig. 3:**
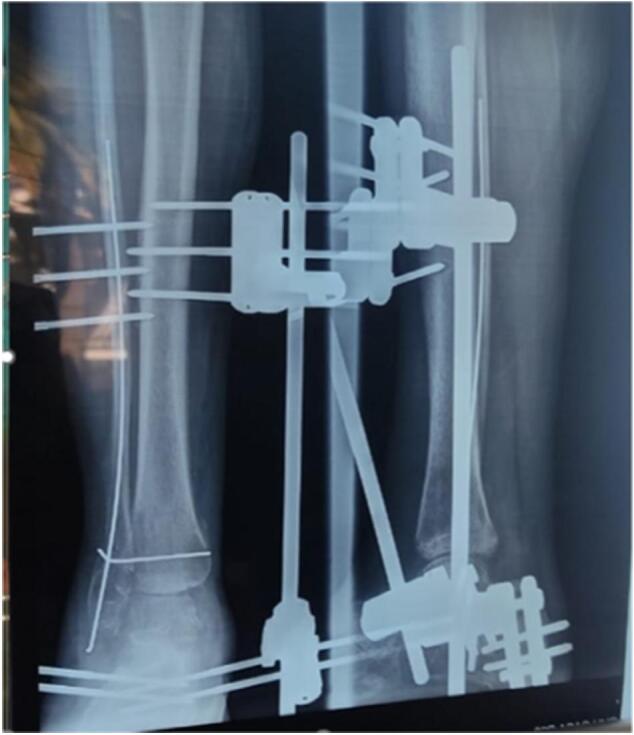
Surgical treatment with k-wiring of the external malleolus, syndesmosis stabilization, and external tibial-calcaneal fixation.

The patient was driven to the operating room where the surgeon realized debridement and abundant irrigation. Surgical exploration found a missing malleolus, without a lesion of the tibialis posterior tendon or the other element of the retro-malleolar groove. The assessment was an external fixation with a tibio-calcaneal frame, k-wiring of the lateral malleolus and insertion of the deltoid ligament into the periosteum of the medial aspect of the tibia (Fig. 3).

**Fig. 4:**
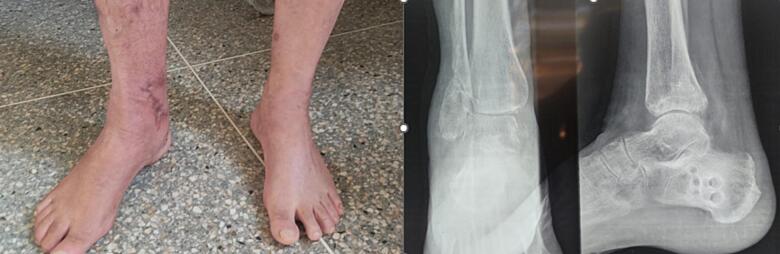
Clinical appearance after a year and the radiologic aspect.

**Fig. 5:**
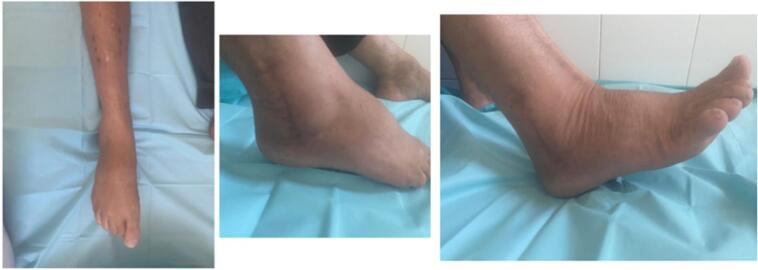
Range of motion of the operated ankle flexion 15°/extension 35°.

The removal of the external fixator was performed after two months. Loading was gradually authorized. The patient was seen in consultation regularly; at one year, the Kaikkonen score [5] was 65 and judged to be fair, with a range of motion of 45° (plantar flexion of 30°- dorsal flexion of 15°), and a satisfaction of the patient was noted (Fig. 4, Fig. 5).

## 3 Discussion

Comprising the distal medial process of the tibia, the medial malleolus articulates with the medial facet of the talus. It is composed of anterior and posterior colliculi, which serve as attachments for the superficial and deep deltoid ligaments, respectively. Whereas the superficial deltoid ligament resists hindfoot eversion, the deep deltoid ligament resists talar external rotation. Medial translation of the talus in the mortise is prevented by the medial malleolus and the anterior and posterior talofibular ligaments [6].

A cadaveric study in the 1970s demonstrated that resection of the lateral malleolus produced significant rotatory and valgus instability. Resection of the medial malleolus, however, resulted in minimal instability [6]. These authors then concluded that an anatomically reduced lateral malleolus is necessary for the successful treatment of bimalleolar fractures and that fixation of the medial malleolus is not essential. Although the talus migrates laterally with the lateral malleolus in bimalleolar ankle fractures, this trend does not occur with isolated lateral malleolar fractures [6].

Authors [[7], [8], [9]] have reported a similar case where there was a posttraumatic missing medial malleolus or surgical excision of the medial malleolus. Periosteal repair of the deltoid ligament was performed. The review noted that the absence of the medial malleolus did not lead to instability. Patient satisfaction was the rule. However, the evolution to tibiotalar arthritis was inevitable. Zhu [10] described the use of an autograft of the iliac crest to ensure reconstruction of the distal malleolus; however, it is not sure to prevent the evolution to a deformity or arthritis.

Misalignment of the talus relative to the tibia is known to alter the joint forces across the ankle joint, even when such misalignment is slight. Older individuals may be less likely to note any deficit from a slight misalignment in the ankle joint. Although little evidence exists to inform this issue [11].

## 4 Conclusion

In our case, there was, as described in the literature, lateral migration of the talus, with good satisfaction of the patient. The absence of the medial malleolus does not lead to instability with a good functional outcome; however, its importance lies in maintaining a normal tibiotalar contact area and pressure. The average decrease in contact area after simulated medial malleolar fractures can range from 27% to 40% with inevitable evolution to ankle arthritis.

## Ethical approval

The study is exempt from ethnical approval in our institution for being just a case report only the patient consent is necessary (comité d'ethique du centre hospitalier IBN Rochd de Casablanca).

## Funding

This research did not receive any specific grant from funding agencies in the public, commercial, or not-for-profit sectors.

## CRediT authorship contribution statement

Dr Foad Lamnaouar: the operator, writing the paper.

Dr Kharroube Mohamed Amine: the aid operator.

Dr Tabbak Khalil following of the patient.

Dr Nekishima Nicos: following of the patient.

Dr Rajaallah Abdssamad reviewing the paper.

Dr Rafai Mohamed: reviewing the paper.

## Guarantor

Dr. Foad Lamnaouar is the guarantor for this study.

## Registration of research studies

None.

## Consent

Written informed consent was obtained from the patient for publication of this case report and accompanying images. A copy of the written consent is available for review by the Editor-in-Chief of this journal on request.

## Declaration of competing interest

The authors declare no conflict of interest.
